# A novel clinically significant prostate cancer prediction system with multiparametric MRI and PSA: P.Z.A. score

**DOI:** 10.1186/s12885-023-11306-2

**Published:** 2023-11-23

**Authors:** Zongxin Chen, Jun Zhang, Di Jin, Xuedong Wei, Feng Qiu, Ximing Wang, Xiaojun Zhao, Jinxian Pu, Jianquan Hou, Yuhua Huang, Chen Huang

**Affiliations:** 1 Department of Urology, The First Affiliated Hospital of Soochow University, 899 pinghai road, Suzhou, 215006 China; 2 Department of Anesthesiology, The First Affiliated Hospital of Soochow University, Suzhou, 215006 China; 3 Department of Radiology, The First Affiliated Hospital of Soochow University, Suzhou, 215006 China; 4 Department of Urology, Dushu Lake Hospital Affiliated to Soochow University, Suzhou, 215000 China

**Keywords:** P.Z.A. score, Clinically significant prostate cancer, Diagnosis, Adjusted prostate specific antigen density of peripheral zone, PIRADS

## Abstract

**Purpose:**

This study aims to establish and validate a new diagnosis model called P.Z.A. score for clinically significant prostate cancer (csPCa).

**Methods:**

The demographic and clinical characteristics of 956 patients were recorded. Age, prostate-specific antigen (PSA), free/total PSA (f/tPSA), PSA density (PSAD), peripheral zone volume ratio (PZ-ratio), and adjusted PSAD of PZ (aPSADPZ) were calculated and subjected to receiver operating characteristic (ROC) curve analysis. The nomogram was established, and discrimination abilities of the new nomogram were verified with a calibration curve and area under the ROC curve (AUC). The clinical benefits of P.Z.A. score were evaluated by decision curve analysis and clinical impact curves. External validation of the model using the validation set was also performed.

**Results:**

The AUCs of aPSADPZ, age, PSA, f/tPSA, PSAD and PZ-ratio were 0.824, 0.672, 0.684, 0.715, 0.792 and 0.717, respectively. The optimal threshold of P.Z.A. score was 0.41. The nomogram displayed excellent net benefit and better overall calibration for predicting the occurrence of csPCa. In addition, the number of patients with csPCa predicted by P.Z.A. score was in good agreement with the actual number of patients with csPCa in the high-risk threshold. The validation set provided better validation of the model.

**Conclusion:**

P.Z.A. score (including PIRADS(P), aPSADPZ(Z) and age(A)) can increase the detection rate of csPCa, which may decrease the risk of misdiagnosis and reduce the number of unnecessary biopsies. P.Z.A. score contains data that is easy to obtain and is worthy of clinical replication.

## Introduction

The incidence of prostate cancer (PCa) has risen dramatically in recent years, making it the second most common male cancer worldwide, affecting approximately 375,000 men per year [1]. Diagnosis of PCa relies on prostate biopsy (PB) [2]. In recent years, multiparameter magnetic resonance imaging (mpMRI) has been increasingly used for diagnosis [3, 4]. In 2015, the American College of Radiologists, the European Society of Urogenital Radiology (EUSR) and the AdMeTech Foundation developed the Prostate Imaging Reporting and Data System (PIRADS) version 2, which was later upgraded to 2.1 [5, 6]. The PIRADS ranges from 1 (clinically significant cancer is highly unlikely to present) to 5 (clinically significant cancer is highly likely to present). Currently, mpMRI is recommended prior to the first PB to improve diagnostic accuracy [7]. The current focus is on identifying patients with clinically significant PCa (csPCa) as early as possible, rather than identifying all PCa [8]. Consequently, a novel csPCa prediction system is being developed to improve diagnostic performance and avoid unnecessary biopsies.

## Patients and methods

### Patient recruitment

In this retrospective cohort study, a total of 1156 male patients presented for PB at The First Affiliated Hospital of Soochow University (Suzhou, China) from June 2016 to August 2021. Of these patients, 63 had previously received treatment, 101 had a prostate-specific antigen (PSA) level above 100 ng ml^− 1^, 36 were unable to undergo MRI examinations, and 956 received a mpMRI examination. MRI-based triaging was performed as described by Donato et al. [9]. Then all of them underwent transperineal PB (TP-PB). Of these, 717 patients from June 2016 to August 2020 were enrolled in the training set, and 239 patients from August 2020 to August 2021 were enrolled in the validation set.

### MRI acquisition

We utilized a 3T MR scanner (MAGNETOM Skyra, Siemens Healthineers, Erlangen, Germany) to acquire images from all patients. The signal was received via an 18channel body and standard spine array coils. The prostate and seminal vesicles were imaged using transverse T1weighted turbo spinecho (TSE) images, as well as the transverse, coronal and sagittal T2weighted TSE images(T2WI). The apparent diffusion was obtained from diffusion-weighted imaging (DWI), which was acquired using a 2-dimensional echo planar imaging sequence with multiple bvalue acquisitions (0, 100 s mm^− 2^, 800 s mm^− 2^, 1000 s mm^− 2^, and 1500 s mm^− 2^), with diffusion-sensitizing gradients applied along the x-, y-, and z-axes. Dynamic contrast enhanced (DCE) imaging was performed using a 3-dimensional(3D) T1weighted gradientecho volumetric interpolated breath-hold examination in the same plane as the 3D T2WI sequence. An intravenous contrast agent (Medtron AG, Saarbruecken, Germany) was then administered at a rate of 1 ml kg^− 1^ body weight and 2.5 ml s^− 1^ injection rate. Finally, the MR Tissue4D software (Syngo. via VA20B; Siemens Healthineers) was used to construct perfusion curves. The method is as described in our previous study [10].

### Prostate biopsy and pathology analysis

TP-PB, including targeted biopsy (TB) and systematic biopsy (SB), was performed on all patients. During TB, the DICOM data of mpMRI images, including T2WI, DWI, apparent diffusion coefficient (ADC) and DCE, were imported into the Real-time Virtual Sonogra (RVS) ultrasonography host (Fujifilm, Japan), and the target lesion was marked as region of interest (ROI). Through RVS, the ROI marked was displayed in real-time on the ultrasonography images. Ultrasonography and MRI images were matched by sagittal and axial anatomical markers, such as urethral orifices and small prostate cysts. Following these steps, the urologist performed the TB, and each ROI was executed on 2-core biopsy. After completing TB, the RVS was turned off, and the same urologist continued to perform SB. All specimens were fixed in 10% formalin and subjected to pathological analysis. The csPCa was defined as a single biopsy core with a Gleason score of 3 + 4 or above (International Society of Urological Pathology (ISUP) grade group (GG) > 1), as previously described [11].

### Patient characteristics

The patients’ age, pre-biopsy PSA, free/total PSA (f/tPSA), and pathological features were included in the study. The included MRI characteristics were PIRADS, prostate volume (PV) on mpMRI (PV = 0.52 × height × length × width), the PSA density (PSAD; PSAD = PSA/PV), transitional zone (TZ) volume (TZV = 0.52 × height-TZ × length-TZ × width-TZ), peripheral zone (PZ) volume (PZV = PV - TZV), PZ-ratio (PZ-ratio = PZV/PV), and aPSADPZ (aPSADPZ = PSAD × PZ-ratio). Each patient was graded according to PIRADS (Version 2.0 was used from June 2016 to January 2020, and Version 2.1 was used from February 2020 to August 2021) by the same radiologist who graded more than 500 prostate MRI readings. The biopsy cores were examined by a dedicated pathologist.

### Statistical analysis

Categorical variables, normal distribution continuous variables and skewed distribution continuous variables were analyzed using the Pearson’s Chi-squared test, T-test and Mann–Whitney U test, respectively. The area under the receiver operating characteristic (ROC) curve (AUC) of individual factors was compared using previously described methods [12]. Binary logistic regression was used to calculate the odds ratios of each predictive factor.

Predictive models were built using the training set by first performing univariate regression analysis to evaluate the power of each parameter in diagnosing csPCa. Next, the variables with a *P*-value < 0.05 and no covariance in the univariate analysis were further analyzed by multivariate logistic regression models using enter selection method. The multivariate regression coefficients were then used to construct nomograms. The P.Z.A. score, which included PIRADS(P), aPSADPZ(Z), and age(A), was used to predict the occurrence of csPCa. The calibration and discrimination abilities of P.Z.A. score were evaluated using the calibration curve and AUC, respectively. The calibration curve was evaluated using both an internal validation cohort (1,000 bootstrap resamples) and an external validation cohort (the validation set). Patients were divided into high-risk and low-risk groups according to the optimal threshold, and the diagnostic efficacy of P.Z.A. score was observed. The clinical benefits of P.Z.A. score were determined by decision curve analysis (DCA). The nomogram, calibration plots, and DCA were constructed using R x64 4.0.2 (R foundation for Statistical Computing, Vienna, Austria, http://www.r-project.org). Other statistical tests were conducted using SPSS v22.0 (IBM Corp, Armonk, NY, USA), MedCalc v18.2.1 (MedCalc Software, Belgium) and Graphpad prism 8.0.2 (Graphpad software, San Diego, CA, USA). All reported P-values were two-sided, and the level of statistical significance was set at P < 0.05.

## Results

### Demographic and clinical characteristics

Overall, 41.8% (400/956) of patients had histologically confirmed csPCa. The clinical data of all patients are summarized in Table 1. Patients with csPCa were significantly older and had higher values of PSAD, PZ-ratio and aPSADPZ, and lower value of f/tPSA and PV, compared to patients with clinically insignificant PCa (cisPCa) or benign disease. The clinical characteristics and pathological results of these patients in the training and validation sets are detailed in Table 1. No significant differences were found in most clinical characteristics between the csPCa and cisPCa/benign disease groups, except for PZ-ratio, which showed no significant difference (P = 0.004).

**Table 1:** Patients demographics and the correlation with biopsy results and enrolled in the training set and the validation set

Characteristic	csPCa (n = 400)	cisPCa or Benign (n = 556)	Z	P	Training set (n = 717)	Validation set (n = 239)	Z	P
Age (year), median (IQR)	72(66–76)	66(61–72)	-9.104	< 0.01	69(63–74)	68(63–75)	-1.013	0.311
PSA (ng ml-1), median (IQR)	15.18(9.36–25.83)	8.76 (5.88–13.82)	-11.336	< 0.01	10.66(6.77–18.83)	11.04 (6.61–16.69)	-0.256	0.798
f/tPSA, median (IQR)	0.104(0.075–0.144)	0.150(0.104–0.206)	-9.695	< 0.01	0.129(0.087–0.181)	0.129(0.081–0.189)	-0.287	0.774
PV (ml), median (IQR)	34.9(26.1–46.5)	48.7(32.0-70.7)	-9.136	< 0.01	41.3(29.6–60.1)	44.0(29.2–70.8)	-1.313	0.189
PSAD (ng ml-2), median (IQR)	0.429(0.240–0.839)	0.175 (0.111–0.280)	-15.444	< 0.01	0.250(0.148–0.469)	0.218 (0.123–0.458)	-1.590	0.112
PZ-ratio, median (IQR)	0.577(0.498–0.666)	0.465(0.345–0.561)	-11.468	< 0.01	0.520(0.407–0.625)	0.478(0.379–0.589)	-2.845	0.004
aPSADPZ (ng ml-2), median (IQR)	0.242(0.135–0.468)	0.076(0.040–0.132)	-17.094	< 0.01	0.125(0.058–0.258)	0.107(0.048–0.241)	-1.770	0.077
PIRADS, n (%)								
2	3(0.8)	178(32.0)	NA	NA	140(19.5)	41(17.2)	NA	NA
3	24(6.0)	250(45.0)	NA	NA	200(27.9)	74(31.0)	NA	NA
4	156(39.0)	90(16.2)	NA	NA	185(25.8)	61(25.5)	NA	NA
5	217(54.2)	38(6.8)	NA	NA	192(26.8)	63(26.4)	NA	NA
ISUP, n (%)								
Benign	0	480(86.3)	NA	NA	363(50.6)	117(49.0)	NA	NA
GG1	0	76(13.7)	NA	NA	49(6.8)	27(11.3)	NA	NA
GG2	154(38.5)	0	NA	NA	107(14.9)	47(19.7)	NA	NA
GG3	120(30.0)	0	NA	NA	95(13.2)	25(10.5)	NA	NA
GG4	66(16.5)	0	NA	NA	48(6.7)	18(7.5)	NA	NA
GG5	60(15.0)	0	NA	NA	55(7.7)	5(2.1)	NA	NA

csPCa: clinically significant prostate cancer; cisPCa: clinically insignificant prostate cancer PSA: prostate specific antigen; f/tPSA: free/total prostate specific antigen; PV: prostate volume; PSAD: prostate specific antigen density; PZ: peripheral zone; aPSADPZ: adjusted prostate specific antigen density of peripheral zone; PIRADS: Prostate Imaging Reporting and Data System; ISUP: International Society of Urological Pathology; GG: grade group; IQR: interquartile range.NA: not available

### ROC curve analysis of predictive factors in comparison with aPSADPZ

**Fig. 1:**
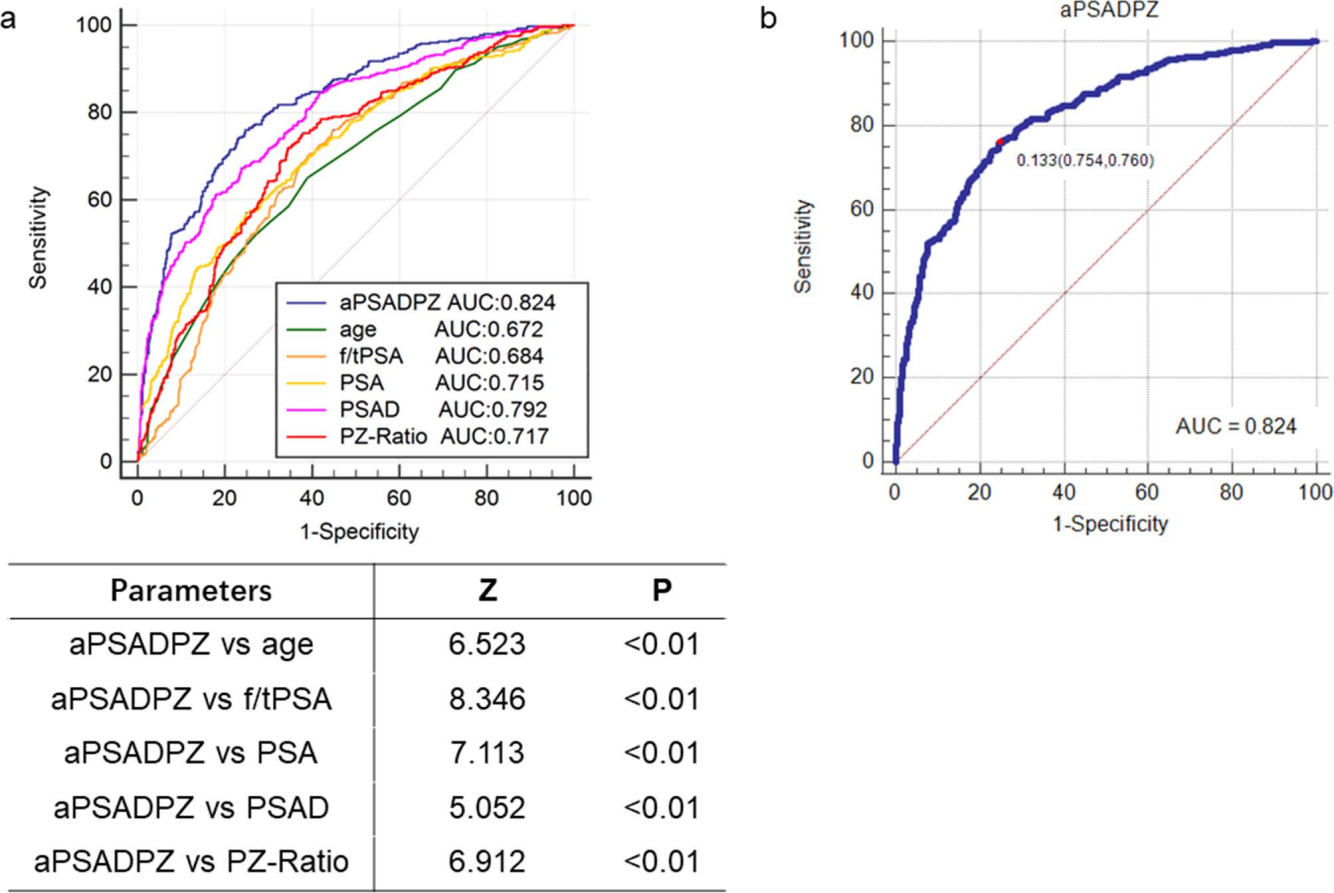
**(a)**ROC curve analysis comparing clinical indicators and their AUCs. **(b)** Optimal threshold, sensitivity, and specificity of aPSADPZ. PSA: prostate specific antigen; f/tPSA: free/total prostate specific antigen; PSAD: prostate specific antigen density; PZ: peripheral zone; aPSADPZ: adjusted prostate specific antigen density of peripheral zone; ROC: receiver operating characteristic; AUC: area under curve

ROC curve analysis revealed that aPSADPZ had highest AUC (0.826) for diagnosing csPCa compared to other parameters such as PSAD (0.792), PZ-ratio (0.717), f/tPSA (0.684), and age (0.672) (Fig. 1a). Pairwise comparisons showed that aPSADPZ had a significantly larger AUC than all other parameters for csPCa diagnosis (aPSADPZ vs. age, Z value: 6.523, P < 0.01; aPSADPZ vs. f/tPSA, Z value: 8.346, P < 0.01; aPSADPZ vs. PSA, Z value: 7.113, P < 0.01; aPSADPZ vs. PSAD, Z value: 5.052, P < 0.01; and aPSADPZ vs. PZ-ratio, Z value: 6.912, P < 0.01). The cut-off for aPSADPZ was 0.133, with a sensitivity of 0.754 and a specificity of 0.760 (Fig. 1b).

### Univariate and multivariate regression analyses of independent predictors for diagnosing csPCa

**Fig. 2:**
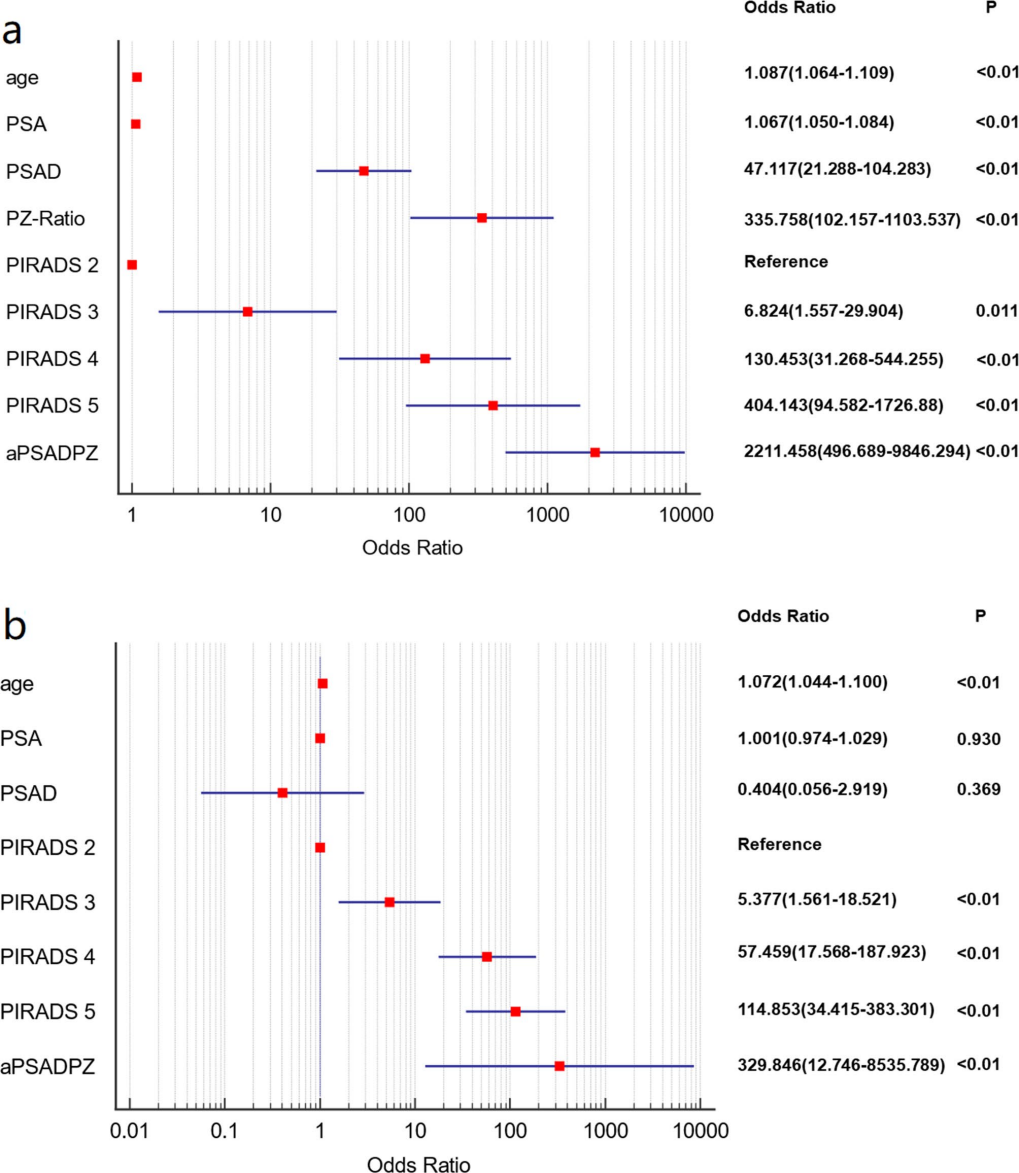
Forest plot of univariate **(a)** and multivariate **(b)** regression analyses for various parameters to detect csPCa. csPCa: clinically significant prostate cancer; PSA: prostate specific antigen; PSAD: prostate specific antigen density; PZ: peripheral zone; aPSADPZ: adjusted prostate specific antigen density of peripheral zone; PIRADS: Prostate Imaging Reporting and Data System

Logistic regression analyses were performed for patients in the training set. As shown in Fig. 2a, age, PSA, f/tPSA, PSAD, PZ-ratio, PIRADS and aPSADPZ were identified as important predictors for diagnosing csPCa in univariate logistic regression analysis. Age, PIRADS and aPSADPZ were found to be significant predictors and were included in the multivariate logistic regression analysis, as shown in Fig. 2b.

### Nomograms and validation of P.Z.A. score for diagnosing csPCa

**Fig. 3:**
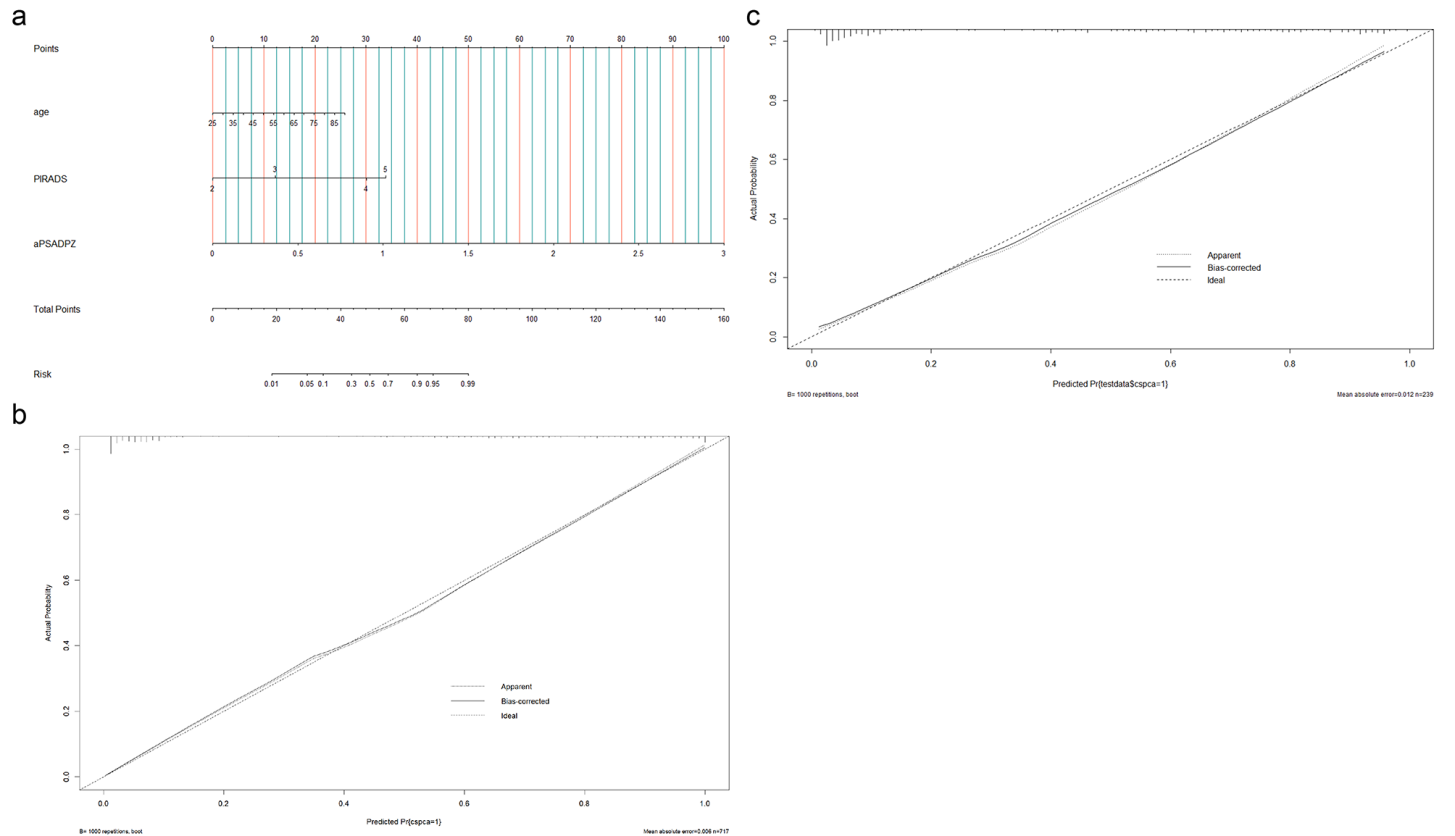
**(a)** Nomogram of P.Z.A. score for predicting the probability of csPCa. Calibration curve of P.Z.A. score in the training set **(b)** and the validation set **(c)**. csPCa: clinically significant prostate cancer; aPSADPZ: adjusted prostate specific antigen density of peripheral zone; PIRADS: Prostate Imaging Reporting and Data System

**Fig. 4:**
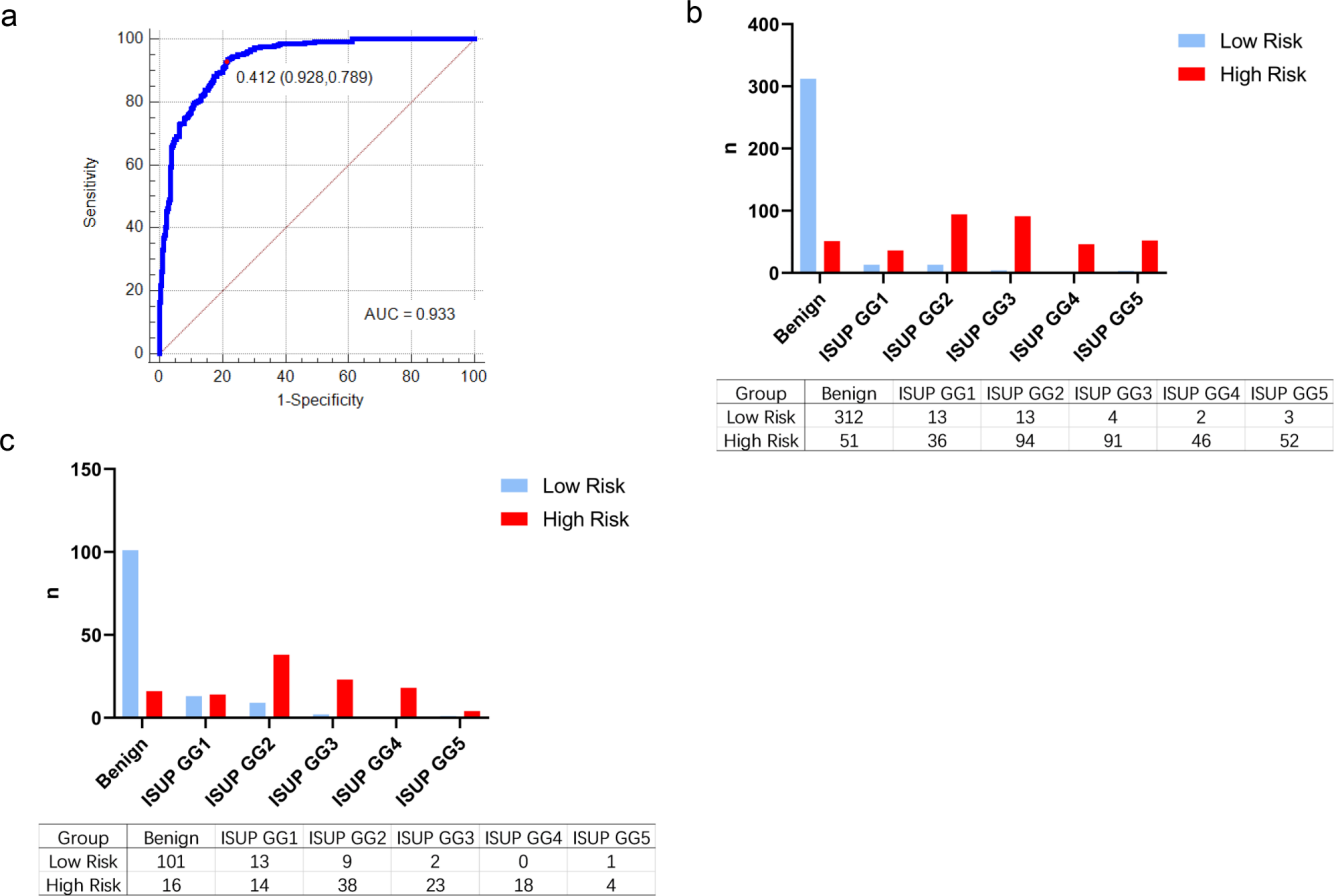
ROC curve analysis, optimal threshold, and the corresponding sensitivity and specificity of P.Z.A. score in the training set **(a)**. Pathological results of low-risk group and high-risk group in the training set **(b)** and the validation set **(c)**. ROC: receiver operating characteristic; ISUP: International Society of Urological Pathology; GG: grade group

Based on multivariate regression coefficients, nomograms (Fig. 3a) were used to visualize predictive results. Calibration curves (Fig. 3b and c) showed excellent calibration between the actual and predicted probabilities of P.Z.A. score for diagnosing csPCa. The AUC of P.Z.A. score was 0.933 with an optimal threshold of 0.41. The corresponding sensitivity and specificity of the prediction model were 0.928 and 0.789, respectively (Fig. 4a). Patients were classified into high-risk and low-risk groups based on the optimal threshold. Pathological results of the two groups were presented in Fig. 4b. Of 22 patients (6.3%) with csPCa, only 5 patients (1.4%) with high-grade (ISUP GG > 3) csPCa (HGPCa) were in the low-risk group. The P.Z.A. score was also applied to patients in the validation set, and patients were classified into high-risk and low-risk groups using a threshold of 0.41.Pathological results of the two groups in the validation set, were shown in Fig. 4c, where 10 patients (7.9%) with csPCa were categorized as the low-risk group, while only 3 patients (2.4%) with HGPCa were in the low-risk group.

### Decision curve analysis for diagnosing csPCa

**Fig. 5:**
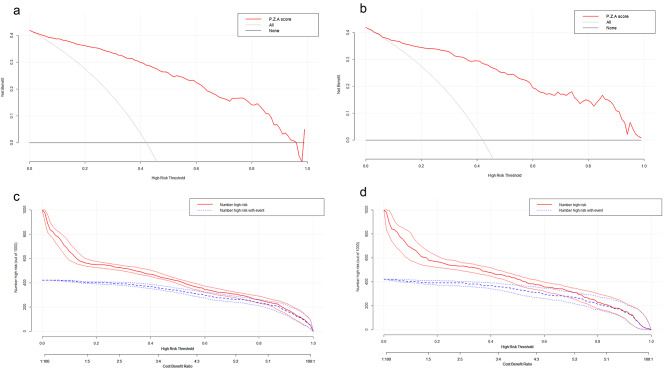
Decision curve analysis of P.Z.A. score for predicting the occurrence of csPCa in the training set **(a)** and the validation set **(b)**. Clinical impact curves of P.Z.A. score for the diagnosis of csPCa in the training set **(c)** and the validation set **(d)**. csPCa: clinically significant prostate cancer

The DCA demonstrated the excellent net benefit of P.Z.A. score in predicting csPCa across all risk threshold in both the training set (Fig. 5a) and validation set (Fig. 5b). In addition, the clinical impact curves indicated that the number of patients with csPCa predicted by P.Z.A. score was in good agreement with the actual number of patients with csPCa in the high-risk threshold, as shown in both the training set (Fig. 5c) and validation set (Fig. 5d).

## Discussion

An ideal detection method for csPCa should have high diagnostic efficacy, less side effects, minimally invasive and reduce cisPCa detection to prevent overtreatment [11]. We propose a diagnostic model that requires a three-step process: model establishment, model validation, and model feasibility analysis. To better address sampling errors and under-sampling during the biopsy process, we have adopted MRI/US fusion TB technology. Multiple studies have shown that TB can detect more csPCa compared to SB [13, 14], mainly because this technology allows for more precise biopsy of lesions identified on MRI, thereby reducing the probability of sampling error and under-sampling.

We use data from the training set for model establishment. The PROMIS study [13] and Satoshi et al. [14] revealed that PIRADS score has a greater significance in the diagnosis of csPCa. We also believe that a pre-biopsy mpMRI is necessary, not only to score the lesion according to PIRADS but also to calculate a series of PV-related parameters from the images. In recent years, several studies have also found that PV-related parameters have some significance in the diagnosis of csPCa. Porcaro et al. [15] found that an increase in the PV index, the ratio of TZV to PZV, reduced the risk of increased tumor load and was associated with a lower biological aggressiveness of PCa in patients randomly biopsied at baseline. Chang et al. [16] showed that the PZ-ratio could also predict csPCa. In contrast, the PSAD calculated from PSA and PV has been used as a predictor of csPCa for many years, as reported by Liang et al. [17] and Luis et al. [18]. We believe that the combination of two indicators, PSAD and a certain PV-related parameter, such as PZ-ratio, can improve the detection rate of csPCa and diagnostic efficacy. In our previous study [10], we performed an initial analysis of aPSADPZ, which combines PSAD and PZ-ratio, and found that aPSADPZ has the largest AUC compared to other parameters. Besides, aPSADPZ is an easily obtainable indicator, requiring only the acquisition of PSA, PV, TZV to calculate. We can use 0.13ng ml^− 2^ as the optimal threshold to facilitate clinical promotion and application.

Eastham et al. [19] reported the first nomogram to predict PCa in 1999. In recent years, several nomograms have been proposed for the diagnosis of csPCa. The P.R.O.S.T. score system established by Liang et al. [16] had high predictive efficacy for csPCa in patients with a PIRADS score of 4 or 5. Zhou et al. [20] found that a nomogram which combined PSA, PV, age and PIRADS can help in clinical decision-making and avoiding unnecessary biopsy. Cabello et al. [21] discovered that the use of PSAD and PIRADS in risk nomograms can provide highly relevant data to increase the accuracy of csPCa diagnosis. In our study, PIRADS(P), aPSADPZ(Z), and age(A) were all important factors in multivariate regression analyses. Therefore, we selected these indicators as a novel model for csPCa diagnosis. For ease of memory, we named this system the P.Z.A. score. We found that the AUC of P.Z.A. score in the training set was 0.933, indicating excellent diagnostic efficacy. We also found the optimal threshold of 0.41for the P.Z.A. score and divided each patient into a high-risk group and a low-risk group based on their risk scores. By comparing the pathological results of the two groups, we observed that the low-risk group had a lower missed diagnosis rate (6.3%), while the high-risk group had a higher detection rate. In particular, for HGPCa, only 1.4% of patients in the low-risk group had the condition. This suggests that the use of the P.Z.A. score can reduce the number of missed diagnoses of csPCa and HGPCa, thus reducing the impact of HGPCa on patients’ lives.

Further, we used DCA to assess the net benefit of using the P.Z.A. score for clinical decision-making. In almost all risk threshold probabilities, the net benefit of the P.Z.A. score was higher than that of “all” or “none”, which is more suitable for guiding clinical decision-making. Moreover, we found good calibration between the actual and predicted probabilities in the region of high probability. Taken together, the P.Z.A. score can improve the detection efficiency of csPCa, avoid unnecessary biopsies and reduce the missed diagnosis of high-risk csPCa. We successfully constructed the predictive model and found it to be highly effective in diagnosing csPCa.

Further validation of P.Z.A. score for diagnosing csPCa in the validation set indicated its excellent performance. First, we found that only 7.9% of csPCa and 2.4% of HGPCa were missed in the low-risk group, while as many as 73.5% of patients were diagnosed with csPCa in the high-risk group. This is important to avoid missing potentially life-threatening cases of csPCa. Moreover, calibration curves drawn from randomly sampled validation set data showed excellent calibration between the actual and predicted probabilities of P.Z.A. score for diagnosing csPCa. The performance of the validation set in DCA was similar to that of the training set, thus verifying that the P.Z.A. score has a good detection rate, a low misdiagnosis rate, and a low miss rate in the diagnosis of csPCa. The data in the model - PSA, age, PV-related parameters are easily accessible, which has a strong clinical application prospect.

In recent years, prostate-specific membrane antigen (PSMA) positron emission tomography (PET) has demonstrated strong efficacy in PCa diagnosis. The PRIMARY study [22] showed that PSMA + MRI improved negative predictive value and sensitivity for csPCa in an MRI triaged population. Donato et al. [23] showed that despite high concordance rates, 68Ga-PSMA PET incrementally improved tumor localization compared with mpMRI. These results suggest that 68Ga-PSMA PET may add value to mpMRI in the diagnostic process for PCa. Scheltema et al. [24] demonstrated that PSMA PET is accurate in detecting segments containing csPCa and is complementary to mpMRI. The combined use of PSMA PET and MRI is a newly proven feasible approach for csPCa diagnosis, providing a theoretical basis for the future integration of PSMA PET into the P.Z.A. scoring system and a viable option for future prospective clinical research.

Our study has several limitations. (1) This was a retrospective and single-institution study performed with a possible risk of selection bias. (2) PIRADS scores are dependent on the experience of a radiologist and may vary from physician to physician. (3) The definition of csPCa used in this study does not include all clinically significant diseases, because ISUP GG1 with high tumor volume load may be significant and ISUP GG2 with low tumor volume load may be insignificant.

In summary, the novel prediction system, P.Z.A. score, can increase the detection rate of csPCa, which may decrease the risk of misdiagnosis and reduce the number of unnecessary biopsies. The P.Z.A. score is based on easily obtainable data and is worthy of clinical replication.

## Data Availability

The datasets used and analyzed during the current study available from the corresponding author upon reasonable request.
